# The Peptidyl-Prolyl *cis-trans* isomerase, Pin1, associates with Protein Kinase C θ *via* a critical Phospho-Thr-Pro motif in the V3 regulatory domain

**DOI:** 10.3389/fimmu.2023.1126464

**Published:** 2023-03-08

**Authors:** Nikhil Ponnoor Anto, Amitha Muraleedharan, Pulak Ranjan Nath, Zuoming Sun, Chen Keasar, Etta Livneh, Alex Braiman, Amnon Altman, Kok-Fai Kong, Noah Isakov

**Affiliations:** ^1^ The Shraga Segal Department of Microbiology, Immunology and Genetics, Faculty of Health Sciences, Ben-Gurion University of the Negev, Beer Sheva, Israel; ^2^ Department of Immunology and Theranostics, Arthur Riggs Diabetes and Metabolism Research Institute, Beckman Research Institute of the City of Hope, Duarte, CA, United States; ^3^ The Department of Computer Science, Ben-Gurion University of the Negev, Beer Sheva, Israel; ^4^ Division of Cell Biology, La Jolla Institute for Immunology, San Diego, CA, United States

**Keywords:** PKCθ, Pin1, protein kinase, prolyl isomerase, T cell activation, immunological synapse

## Abstract

Protein kinase C-θ (PKCθ) is a member of the novel PKC subfamily known for its selective and predominant expression in T lymphocytes where it regulates essential functions required for T cell activation and proliferation. Our previous studies provided a mechanistic explanation for the recruitment of PKCθ to the center of the immunological synapse (IS) by demonstrating that a proline-rich (PR) motif within the V3 region in the regulatory domain of PKCθ is necessary and sufficient for PKCθ IS localization and function. Herein, we highlight the importance of Thr^335^-Pro residue in the PR motif, the phosphorylation of which is key in the activation of PKCθ and its subsequent IS localization. We demonstrate that the phospho-Thr^335^-Pro motif serves as a putative binding site for the peptidyl-prolyl *cis-trans* isomerase (PPIase), Pin1, an enzyme that specifically recognizes peptide bonds at phospho-Ser/Thr-Pro motifs. Binding assays revealed that mutagenesis of PKCθ-Thr^335^-to-Ala abolished the ability of PKCθ to interact with Pin1, while Thr^335^ replacement by a Glu phosphomimetic, restored PKCθ binding to Pin1, suggesting that Pin1-PKCθ association is contingent upon the phosphorylation of the PKCθ-Thr^335^-Pro motif. Similarly, the Pin1 mutant, R^17^A, failed to associate with PKCθ, suggesting that the integrity of the Pin1 N-terminal WW domain is a requisite for Pin1-PKCθ interaction. *In silico* docking studies underpinned the role of critical residues in the Pin1-WW domain and the PKCθ phospho-Thr^335^-Pro motif, to form a stable interaction between Pin1 and PKCθ. Furthermore, TCR crosslinking in human Jurkat T cells and C57BL/6J mouse-derived splenic T cells promoted a rapid and transient formation of Pin1-PKCθ complexes, which followed a T cell activation-dependent temporal kinetic, suggesting a role for Pin1 in PKCθ-dependent early activation events in TCR-triggered T cells. PPIases that belong to other subfamilies, i.e., cyclophilin A or FK506-binding protein, failed to associate with PKCθ, indicating the specificity of the Pin1-PKCθ association. Fluorescent cell staining and imaging analyses demonstrated that TCR/CD3 triggering promotes the colocalization of PKCθ and Pin1 at the cell membrane. Furthermore, interaction of influenza hemagglutinin peptide (HA^307-319^)-specific T cells with antigen-fed antigen presenting cells (APCs) led to colocalization of PKCθ and Pin1 at the center of the IS. Together, we point to an uncovered function for the Thr^335^-Pro motif within the PKCθ-V3 regulatory domain to serve as a priming site for its activation upon phosphorylation and highlight its tenability to serve as a regulatory site for the Pin1 *cis-trans* isomerase.

## Graphical Abstract

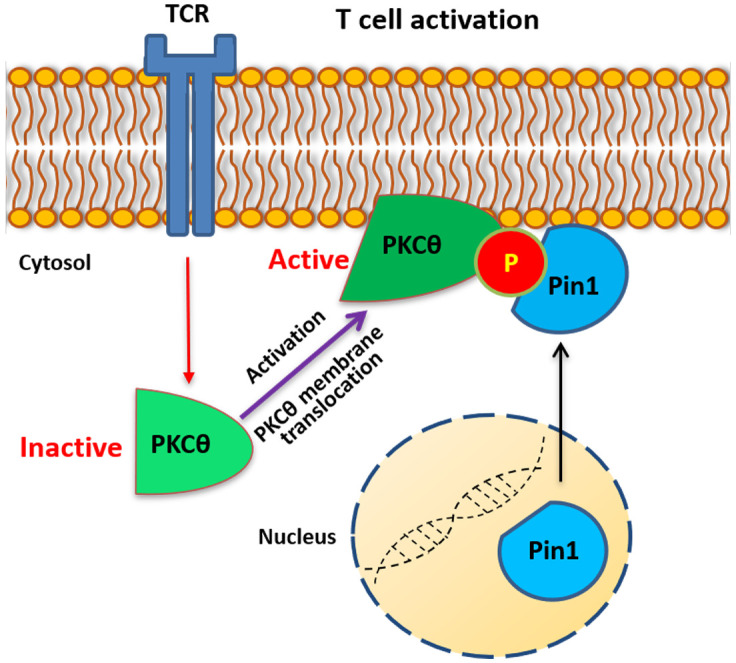

## Introduction

Protein kinase C θ (PKCθ) is highly expressed in T lymphocytes and is *sine qua non* for T cell antigen receptor (TCR)-coupled signal transduction which eventually leads to cell activation and proliferation. Importantly, PKCθ is non-redundant with other T cell-expressed PKC isoforms and is unique in its ability to translocate to a defined central sub-domain of the immunological synapse (IS) in antigen-stimulated T cells (1–6). Signals delivered by the TCR and the CD28 costimulatory receptor induce membrane translocation and kinase activation of PKCθ (7). As a critical component of the TCR/CD28-coupled signaling machinery, PKCθ has the ability to integrate multiple signals and fine-tune signaling pathways required for a specific output. It is subjected to regulation by multiple mechanisms and undergoes phosphorylation at several sites that determine its spatiotemporal conformation and catalytic activity, as well as its ability to interact with cofactors, substrates, and other binding proteins. Although the TCR stimulation-induced PKCθ recruitment to the IS is well established (4, 8), the underlying molecular basis has remained elusive for a long time. Hitherto, we provided a mechanistic explanation for this selective process by showing that the PKCθ-V3 polyproline-rich region, a phylogenetically conserved sequence across species (9), is involved in the localization of PKCθ to the center of the IS, which is mediated by indirect association with the cytoplasmic tail of the costimulatory receptor CD28 (9). Despite the importance of PKCθ in T cell activation, the entire spectrum of mechanisms that regulate the kinase is still lacking.

Proline-directed protein phosphorylation has a crucial role in diverse cellular processes and signal transduction pathways. It is one of the most frequent post-translational modification sites in proteins (10). Proline exists in one of two distinct forms that acquire either *cis* or *trans* conformations. The *cis*-*trans* interconversion of Pro in peptides can occur spontaneously in an aqueous solution at a very slow rate. However, peptidylprolyl isomerases (PPIases) can rapidly interconvert the *cis*-*trans* isomers of peptide bonds with the amino acid proline. PPIases can modulate protein topology by catalyzing a *cis-trans* switch at appropriate Xxx-Pro motifs (where Xxx indicates any amino acid), thereby regulating the reversible transition of substrate proteins between active and nonactive isomers (11). The PPIases are grouped into three families namely: cyclophilins, FK506-binding proteins (FKBPs), and parvulins. These enzymes assist in the folding of newly synthesized proteins and regulate the stability, localization, and activity of mature proteins. For instance, works from our lab have established PPIases regulation on signaling proteins CT-10 regulator of kinase (Crk) and zeta chain associated protein (ZAP-70) (12–14). They also regulate gene transcription at multiple levels. The phospho-Ser/Thr-Pro (pS/T-P) motifs in proteins are thermodynamically hindered and render inaccessible to conventional PPIases, such as cyclophilins and FKBPs. In contrast, the pS/T-P bond serves as a specific recognition motif for Pin1, a member of the parvulin family, which can *cis/trans* isomerize the proteins and regulate their stability and activity (15). Pin1 regulates the activity of various transcription factors that are essential for T cell activation, including NF-κB and AP1. It also regulates the activity of protein kinases and phosphatases at the plasma membrane, cytosol, and nucleus (16, 17). A report by Abrahamsen et al. suggests that Pin1 provides a ‘timer’ for the lifetime of conventional PKC (cPKC) isoforms by converting them into a species that can be readily dephosphorylated, ubiquitinated, and degraded following cell activation by agonists (18). In these studies, the regulation by Pin1 required both the catalytic domain of the isomerase and the presence of a Pro immediately following the phosphorylated Thr at the turn-motif phosphorylation site, one of two C-terminal sites that are phosphorylated during the maturation of PKC isozymes (18).

Human PKCθ possesses seven Ser/Thr-Pro motifs that theoretically, upon phosphorylation, could serve as putative Pin1-binding or -regulatory sites. In the present work, we demonstrate the significance of the phospho-Thr^335^-Pro motif within the V3 polyproline-rich region of the PKCθ regulatory domain, which functions as a critical priming site for the activation of PKCθ, its binding to CD28 and subsequent IS localization. We highlight the potential role of the TCR stimulation-induced transient phosphorylation of the Thr^335^-Pro motif in the spatiotemporal regulation of PKCθ and in the temporal association between PKCθ and the PPIase Pin1. TCR/CD3 crosslinking or co-culture of antigen-specific T cells with antigen-pulsed APCs led to the colocalization of PKCθ and Pin1 at the T cell membrane or at the IS, respectively. Together, we portray Pin1 as a novel binding partner of PKCθ in activated T cells.

## Materials and methods

### Antibodies and reagents

Mouse monoclonal antibodies (mAbs) directed against PKCθ (#610090), and hamster mAbs directed against CD3ϵ (145-2C11, #550275) were from BD Transduction Laboratories (Lexington, KY). Hamster mAbs directed against mouse CD28 (clone 37.51; #102102) were from Biolegend (San Diego, CA). Rabbit anti-Pin1 (#76309) mAbs and rabbit anti-FK506 binding protein 12 (FKBP12) (#92459) mAbs, rabbit anti-IgG isotype control mAbs (#172730) and goat polyclonal anti-mouse IgG (#6708) Abs, Goat anti-hamster IgG (#5738) Abs and CytoPainter Cell Staining Reagent were from Abcam (Cambridge, UK). Rabbit anti-Cyclophilin A mAb (CypA; #133494) was from Santa Cruz Biotechnology, Inc. Horseradish peroxidase (HRP)-conjugated sheep anti-mouse, donkey anti-rabbit and Goat-anti-hamster immunoglobulin Abs, ECL (Electrochemiluminescence), glutathione-coupled sepharose beads, protein A-, G-, and A/G-agarose beads, and nitrocellulose membranes were from Amersham Pharmacia Biotech (Uppsala, Sweden). Mouse anti-β actin (clone AC-15) mAb were from Sigma-Aldrich. Mouse anti-human CD3ϵ (OKT3) and anti-c-Myc mAbs (clone 9E10) were prepared as previously described (19). Protease inhibitor cocktail tablets were from Roche (Basel, Switzerland). Mouse anti-Xpress (#R910-25), Alexa Fluor™ 488-, 546- and 633-conjugated rabbit and mouse Ig Abs, goat anti-mouse Ig Abs, and Alexa Fluor™ 633-conjugated phalloidin were from Thermo Scientific, Inc. (Waltham, MA). Molecular weight marker (Page Ruler) was from Thermo Fisher Scientific. Tricine sample buffer (#161-0739) was from Bio-Rad Laboratories, Inc. Ampicillin was from AppliChem Inc. (Maryland Heights, MO). Chemically competent DH5α™ cells were from Invitrogen (Carlsbad, CA). Calf intestinal alkaline phosphatase (CIP) and lambda phosphatase were from New England Biolabs, Inc. (Ipswich, MA).

### Animals, cell lines, culture conditions, and cell stimulation

Spleen and thymus cells were obtained from 6-8-wk-old C57BL/6J female mice that were housed under controlled conditions with 12 h light/dark cycle and free access to food and water. Splenic and thymic T cells were obtained by density gradient centrifugation using Ficoll-Paque™ solution (Thermo Fisher Scientific). Wild-type C57BL/6 mice were purchased from Harlan. This study was approved in advance by the Ben-Gurion University Institutional Animal Care and Use Committee and conducted in accordance with the Israeli Animal Welfare Act following the guidelines of the Guide for *Care and Use of Laboratory Animals* (National Research Council 1996). The Animal ethical clearance protocol used for this research is IL-44-08-2017.

Cell lines used in this study include the human T cell leukemia, Jurkat (clone E6-1; ATCC^®^ TIB-152™), Jurkat Tag, which stably expresses the Simian Vacuolating Virus 40 (SV40)-derived large T Ag, TCR-deficient Jurkat T cell line, CH7C17, which was transfected with the influenza hemagglutinin (HA^307-319^) peptide-specific, DR1-restricted HA1.7 TCR, and the EBV-transformed human B cell line, LG2, which expresses the HLA-DR1 and B7.1 surface receptors, human Burkitt’s lymphoma line, Raji, and African green monkey kidney-derived COS-7 cells. Jurkat cell lines were maintained at a logarithmic growth phase in RPMI (Sigma), supplemented with 10% heat-inactivated fetal calf serum (FCS), 2 mM L-glutamine, 50 U/ml penicillin and 50 μg/ml streptomycin (all from Biological Industries, Kibbutz Beit Haemek, Israel)) (complete RPMI), and grown in 75-cm^2^ tissue culture flasks (CellStar, Greiner, Germany). COS-7 cells were maintained at a logarithmic growth phase in DMEM (Sigma) supplemented with 10% heat-inactivated FCS, 50 units/ml penicillin, and 50 μg/ml streptomycin (complete DMEM) in 145 mm tissue culture plates (CellStar, Greiner, Germany).

Jurkat T cell stimulation *via* the TCR was performed by incubation with anti-CD3 mAbs (OKT3, 30 min on ice) plus cross-linking with a secondary Ab, goat anti-mouse IgG, for the indicated time intervals, at 37°C. TCR stimulation of mouse spleen and thymus cells was carried out using hamster anti-CD3ϵ mAbs (145-2C11, 30 min on ice) plus cross-linking with a secondary Ab, goat anti-hamster IgG, for the indicated time intervals, at 37°C.

### Expression vectors, peptides, and site-directed mutagenesis

pGEX bacterial expression vectors encoding GST-Pin1, GST-Pin1-C113A, and GST-Pin1-R17A were gifts of Hung-Ying Kao (Case Western Reserve University). Luciferase reporter constructs for NFAT, NF-κB, CD28 response element RE/AP, and the β-Galactosidase (β-Gal) reporter plasmids were obtained from M. Karin. Empty vector or expression vectors for Xpress-tagged wild-type [WT (T^335^P)], or mutant PKCθ (ΔV1, ΔV3, ΔV4, and ΔV6) were prepared as previously described (9).

Myc-tagged peptides, KS21 and pKS21 peptides, which include the PKCθ-V3 domain-derived Thr^335^-Pro motif plus ten amino acids on either side of this sequence were from GL Biochem (Shanghai) Ltd. The KS21–Myc peptide (KNEARPPCLPTPGKREPQGIS-EQKLISEEDL) lacks a phosphoryl group on Thr^335^ while pKS21-Myc (KNEARPPCLP(pT)PGKREPQGIS-EQKLISEEDL) includes a phosphoryl group on Thr^335^.

PKCθ mutants [T/A, A^335^P; T/E, E^335^P] were generated by site-directed mutagenesis and overlapping PCR.

### Nucleofection of Jurkat T cells, and transient transfection of eukaryotic cells

Non-adherent cells: Jurkat T cells maintained at a logarithmic phase were washed in supplement-free RPMI 1640, resuspended either in 700 μl transfection buffer (1.8 g/ml sucrose, 2nM DTT in RPMI 1640) or in 250 μl Mirus Ingenio^®^ electroporation solution and transferred into 0.4 cm-gap Gene Pulser cuvettes (BioRad) (20x10^6^ cell/700 μl/cuvette). Plasmid DNA (10 μg/group, unless otherwise indicated) was added to the cuvettes, and electroporation was performed using a BioRad Gene Pulser (250V, 950mF). The cells were then cultured in 50 ml of complete RPMI 1640 in 145 mm tissue culture plates for 48 h. Unless otherwise indicated, all nucleofection experiments were carried out in triplicates using 3 separate dishes for each point.

Adherent cells: COS-7 cells maintained at a logarithmic phase were collected from culture dishes by trypsin treatment, washed in supplement-free DMEM, resuspended in 5 ml of 10% FCS-containing DMEM (2x10^6^ cells/ml), and cultured in CellStar tissue culture flasks (Greiner Bio-one 690170). Within 24 hours of plating, adherent cells were transfected with the indicated DNA (5μg/group, unless otherwise indicated) using the PEI (Polysciences) transfection reagent in a 1:3 (DNA: PEI) ratio. The cells were then cultured in 10 ml complete DMEM in 75 cm^2^ tissue culture flasks for 48h.

### Cell lysate preparation, immunoprecipitation, and immunoblotting

Cell lysates were prepared by resuspension of cells in a lysis buffer (25 mM Tris-HCl, pH 7.5, 150 mM NaCl, 5 mM EDTA, 1 mM Na_3_VO_4_, protease inhibitor cocktail (1 tab/10 ml), and 1% Triton X-100), followed by a 30 min incubation on ice. Lysates were centrifuged at 13,000xg for 30 min at 4°C and the nuclear-free supernatants were used for immunoprecipitation. Immunoprecipitation was performed by pre-adsorption of primary Abs onto protein A-, G-, or protein A/G-coupled beads, based on the differential affinities of the proteins to specific Abs, for 2 h at 4°C. Equal volumes of 2x Laemmli Sample Buffer were added to immunoprecipitates or whole-cell lysates, which were vortexed, boiled for 5 min, and fractionated by SDS-PAGE, either on 10%, or 12.5% polyacrylamide gels, and 16.5% Tris-Tricine gels, using Bio-Rad Mini-PROTEAN II cells. Proteins from the gel were electroblotted onto nitrocellulose membranes (Schleicher and Schuell) at 100V for 1 h, using BioRad Mini Trans-Blot transfer cells. After 1 h of blocking with 3% BSA in TBST at 37°C, the nitrocellulose membranes were incubated in the presence of the indicated primary Abs, followed by incubation with HRP-conjugated secondary Abs. Immunoreactive protein bands were visualized using an ECL reagent and autoradiography.

### GST fusion proteins and pull-down assay

pGEX bacterial expression vectors were used to transform *Escherichia Coli* DH5α™ competent cells and GST-fusion proteins were prepared as described (20). Pull-down assays were performed by incubation of bead-immobilized GST or GST fusion proteins (2-5 μg/sample) with cell lysates at 4°C on a rotator for 4 h. The beads were washed (x3) in lysis buffer and bound proteins were eluted and subjected to SDS-PAGE under reducing conditions, followed by immunoblotting.

### Preparation of mouse thymus tissue sections

Thymi from 6-8-wk-old C57BL/6J mice were immersed in a 4% paraformaldehyde solution at 4°C overnight and embedded into paraffin blocks that were cut into 4-6 µm slices. The tissue sections were mounted on positively-charged glass slides and dried for 1 h at 60°C, deparaffinized in xylene, and rehydrated in absolute ethanol. The slides underwent an antigen retrieval step in sodium citrate buffer (pH 6) in a steam pot. After boiling, sections were kept for cooling at room temperature for 30 min.

### Immunofluorescence staining and confocal microscopy

Jurkat T cells (2×10^6^/group) were seeded on poly-L-lysine-coated 8 well µ-slide (ibidi Ltd.) and deposited on the slides by centrifugation at 1200 rpm for 5 min. Cells were fixed in 4% paraformaldehyde (Sigma)/PBS for 5 min at room temperature, permeabilized with PBS/0.1% Triton X-100 for 5 min, and blocked with Ab Diluent blocking solution (GBI Labs) for 1 h at RT. Cells were then incubated with mouse anti-PKCθ mAb and rabbit anti-Pin1 mAb in blocking buffer for 1 h at room temperature. After three washes in PBS, the cells were incubated with Alexa Fluor 546-conjugated anti-mouse (or rabbit) Ig and Alexa Fluor 488-conjugated anti-rabbit (or mouse) Ig secondary Abs for 1 h in the dark at RT and counterstained with DAPI (1µg/ml) in PBS for 15 min. Staining specificity was tested by parallel cell treatment with the same reagents, except for the primary Abs.

Thymus sections were incubated with 3% normal goat serum (1 h), followed by incubation with goat anti-mouse IgG (1 h in a humidified chamber at room temperature) to block nonspecific binding. After careful removal of excess serum, the sections were overlaid with mouse anti-PKCθ mAbs and rabbit anti-Pin1 mAbs for 16 h in a humidified chamber at 4°C. The sections were washed in PBS and incubated with secondary Abs plus DAPI, as above. A control experiment was performed by staining of C57BL/6J mouse thymic sections with labeled Alexa Fluor™546-conjugated goat anti-mouse and Alexa Fluor™488-conjugated goat anti-rabbit secondary Abs, in order to rule out the possible basal, and non-specific staining. The coverslips were mounted on slides using DAKO mounting medium and imaged by Olympus FluoView FV1000 laser-scanning confocal microscope. Immunofluorescence images represent single 2-D confocal sections. The extent of colocalization of Pin1 and PKCθ was quantified using the *JACoP ImageJ plugin* (21).

### Conjugate formation assay and immunofluorescence

Immunological synapse studies were performed as previously described (22). Jurkat E6.1 T cells transfected with an expression vector for Xpress-tagged wild-type or mutant PKCθ, were incubated at a ratio of 1:1 with Raji B cells pre-pulsed with Staphylococcus enterotoxin E (SEE) for 5 min. After fixation, conjugates were stained with mouse anti-Xpress plus a secondary Alexa fluor 488-coupled Ab (AF488) and counterstained with DAPI. Similarly, Jurkat-CH7C17 T cells, which express the influenza hemagglutinin (HA^307-319^) specific TCR, were incubated with peptide-loaded antigen-presenting cells (APCs), LG2, at a ratio of 1:2 at 37°C for 5 min. APCs were pre-loaded with 200 μg/ml of HA^307-319^ peptide, or an inactive HA peptide, in which Lys316 was replaced by Glu (K316E), for 3 hours at 37°C. LG2 cells were prestained using CytoPainter Cell Staining Reagent prior to the cell mixing. Following 5 min of co-incubation, the cells were fixed, permeabilized, and analyzed by confocal microscopy. Immunofluorescence staining was performed using the primary Abs: mouse anti-PKCθ and rabbit anti-Pin1, followed by incubation with Alexa Fluor 633-conjugated phalloidin and: Alexa Fluor 488-conjugated anti-rabbit IgG and Alexa Fluor 546-conjugated anti-mouse IgG. CytoPainter was detected at 405nm (Olympus FluoView FV1000 laser-scanning confocal microscope).

### *In silico* modeling of the proposed PKCθ-Pin1 complex

Using the Swiss-PdbViewer package (23), we modeled the complex of the WW domain of Pin1 with PKCθ using the structure of another Pin1 complex as a template. The crystal structure of Pin1 bound to a phosphorylated peptide, via its WW domain, was solved by Verdecia et al. (PDB code 1F9A) (24). The peptide in that structure, which is derived from RNA polymerase II, is Tyr-pSer-Pro-Thr-pSer^174^-Pro-Ser. While the crystal structure also includes the catalytic domain of Pin1, the domain interface is rather small, and does not include the peptide. Thus, our modeling includes only the WW domain of Pin1. We threaded the Pro-Leu-Pro-Thr^335^-Pro-Gly peptide of PKCθ on the bound peptide, with pSer^174^-Pro and pThr^335^-Pro respectively as anchors. The major binding pocket in the crystal structure includes Ser^16^, Arg^17^, and Ser^18^ that coordinate the phosphate group and Trp^34^ and Tyr^23^ that line a hydrophobic pocket that fit the proline side chain. The corresponding PKCθ moieties fit this pocket in the model by construction, as they adopt the coordinates of almost identical residues. Tyr^23^ also lines a minor binding pocket, together with Phe^25^. In the crystal structure this pocket is occupied by a proline side chain, in the model it snugly fit a Leucine side chain, after adjustment of side-chain rotamers. All the other peptide residues hardly contact the WW domain, and thus replacing them with by the PKCθ residues in the model does not confer any clashes or incompatibility.

### Retroviral shRNA silencing of Pin1 in Jurkat T cells

Plasmids for silencing Pin1 gene, pSuper-retro-puro-shPin1, and a control vector, pSuper-retro-puro-shControl were gifts of Prof. Akihide Ryo, Yokohama City University: (pSUPER RNAi System, Oligoengine). A mix containing a packaging plasmid, pPAX2 (900 ng), an envelope plasmid pVSV-G (100 ng), and the selected pSuper-retro-puro-shPin1 or pSuper-retro-puro-shControl (1 μg) was prepared in OPTI-MEM serum-free media (Invitrogen, #31985-070) The plasmid mix was added to an LT mix containing Transit IT^®^-LT1 transfection reagent and Opti-MEM. The transfection mix was added to the HEK293T packaging cells, which were maintained in DMEM containing 10% FBS and 1% Pen-Strep. On the next day, the medium was replaced by adding complete fresh DMEM medium. After an additional day, the medium was collected and spun at 1,250 rpm for 5 minutes. Polybrene (8µg/ml) was added to the medium, which was then filtered through a 0.44µm filter and used for transfection of Jurkat cells. The Jurkat cells (2x10^5^) were incubated with the transfection medium for 5 hours, and Pin1 gene-silenced cells were obtained by cell growth for 48 hours in a selection medium containing various concentrations of puromycin.

### Statistical analysis

Statistical analysis was carried out using GraphPad Prism software Version 9.2.0. Microscopy data were quantified using the ImageJ plugin, *JACoP.* Statistical significance of differences between groups of averaged data points was assessed using the One-way ANOVA (Tukey’s Multiple Comparison test) or Two-way ANOVA as indicated.

## Results

### Phosphorylation of Thr^335^-Pro is critical for PKCθ activation, binding to partners, and IS localization

**Figure 1:**
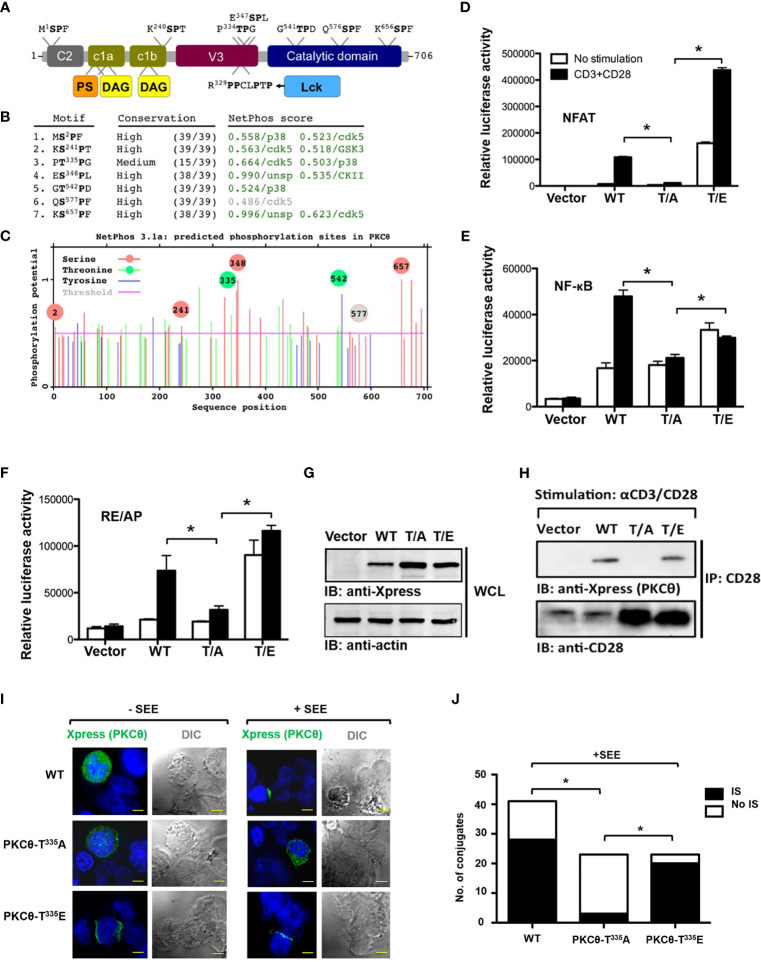
Phosphorylation of Thr^335^-Pro is required for PKCθ activation, binding to CD28, and IS localization. **(A)** The modular structure of PKCθ and some of its potential functional motifs. PKCθ possesses an N-terminal regulatory region with several conserved functional domains and a C-terminal catalytic region. A recently described polyproline-rich region within the V3 domain mediates CD28 binding, *via* Lck, as an intermediate protein, and controls PKCθ recruitment to the center of the immunological synapse. Short independent sequences containing Ser/Thr-Pro motif in human PKCθ are indicated above the figure representing the PKCθ protein backbone. Phosphorylation of each of these Ser/Thr residues can potentially serve as Pin1 binding/isomerization sites. **(B)** Human PKCθ protein sequence analysis, using NetPhos 3.1, predicted six out of seven Ser/Thr-Pro motifs as *in vivo* phosphorylation sites (score values >0.5, green color), and made kinase-specific predictions for the majority of these sites. Unsp=unspecified kinase. **(C)** A diagram showing sequence positions of all NetPhos 3.1-predicted phosphorylation sites on PKCθ. Phosphorylation sites within Ser/Thr-Pro motifs are indicated by a circle (Ser-red; Thr-green)(grey-below the threshold value), including positions of the putative phosphoresidues.**(D–G)** Luciferase activity in Jurkat E6.1 T cells transfected with expression vectors for Xpress-tagged wild-type [WT (T^335^P)], mutant PKCθ [T/A, A^335^P; T/E, E^335^P] or an empty vector, together with a luciferase reporter for NFAT **(D)**, NF-κB **(E)**, or the CD28 response element RE/AP **(F)**, and β-Galactosidase (β-Gal) reporter, post-stimulation with anti-CD3 plus anti-CD28 mAbs. Results are presented as relative units (RLU) of luciferase to β-Gal activities. *P<0.05 (one-way ANOVA). Whole cell lysate immunoblot analysis of PKCθ expression probed with anti-Xpress, and anti-β-actin, as a protein loading control **(G)**. **(H)** Analysis of PKCθ coimmunoprecipitation with CD28 from lysates of Jurkat E6.1 T cells transfected with the indicated Xpress-tagged PKCθ vectors or an empty vector, and stimulated for 5 min with anti-CD3 plus anti-CD28 mAbs. Immunoblot (IB), immunoprecipitates (IP). **(I)** Immunofluorescence imaging of Jurkat E6.1 T cells that were transfected with an expression vector for Xpress-tagged wild-type or mutant PKCθ and incubated at a ratio of 1:1 with Raji B cells pulsed with Staphylococcus enterotoxin E (SEE) for 5 min. After fixation, conjugates were stained with mouse anti-Xpress plus a secondary Alexa fluor 488-coupled Ab (AF488) and counterstained with DAPI (blue, nuclear staining). Original magnification, x 60, Scale bar, 2μm. Data are representative of three experiments. DIC, Differential interference contrast. **(J)** Quantification of the localization of PKCθ in (IS) or outside (No IS) the immunological synapse and cSMAC in T cell-APC conjugates as described in I (n= 23-40), limited to conjugates that had reorganized their talin and had detectable PKCθ. *P<0.05 (one-way analysis of variance [ANOVA)].

PKCθ possesses an N-terminal regulatory region with several conserved functional domains and a C-terminal catalytic region. A proline-rich (PR) motif within the V3 region in the PKCθ regulatory domain was recently found to be necessary and sufficient for PKCθ IS localization and function (9). Moreover, human PKCθ harbors several Ser/Thr-Pro motifs that are predicted to be phosphorable, posing them as additional potential regulatory sites. Among them, Thr^335^-Pro was of particular interest, owing to its location within the unique PR hinge region of PKCθ (**Figures 1A–C**). To test this conjecture, mutagenesis of this key Thr^335^ residue, followed by PKCθ-related functional aspects has been employed. Luciferase reporter assay and coimmunoprecipitation studies were utilized to test whether Thr^335^ plays a role in PKCθ activation and/or ability to interact with binding partners. Jurkat T cells were utilized for both assays following their nucleofection with wild-type or the Thr^335^ point mutants [T/A, A^335^P; T/E, E^335^P]. For the reporter assay, the cells were cotransfected with a luciferase reporter for PKCθ-downstream effector molecules, including NFAT and NF-κB, or the CD28 response element RE/AP, and tested following TCR crosslinking using anti-CD3/CD28 mAbs. For the binding assay, PKCθ wild-type or the Thr^335^ mutant-overexpressing Jurkat T cells were lysed and subjected for coimmunoprecipitation using anti-CD28 mAbs. Our results demonstrated that phosphorylation of Thr^335^ is required for the activation of PKCθ (**Figures 1D–G**) and its binding to the costimulatory receptor, CD28 (**Figure 1H**). Thr^335^-to-Ala amino acid substitution completely abolished the ability of PKCθ to be triggered or to bind CD28, while replacement of Thr^335^ by Glu, which mimics the approximate size and charge of phospho-Thr, restored these functions. Furthermore, a conjugate formation assay, utilizing Jurkat T cells, Raji B cells as APCs, and staphylococcal enterotoxin (SEE) as a TCR stimulant, followed by fluorescent cell staining and confocal microscopy confirmed that phospho-Thr^335^ is required for PKCθ localization at the IS (**Figures 1I, J**). These data highlight the potential role of the Thr^335^-Pro motif in the spatiotemporal regulation of PKCθ and suggest that regulation *via* this motif is phosphorylation-dependent.

### Pin1 mediates a phosphorylation-dependent binding of PKCθ, and the integrity of the PKCθ-V3 is a requisite for this interaction

**Figure 2:**
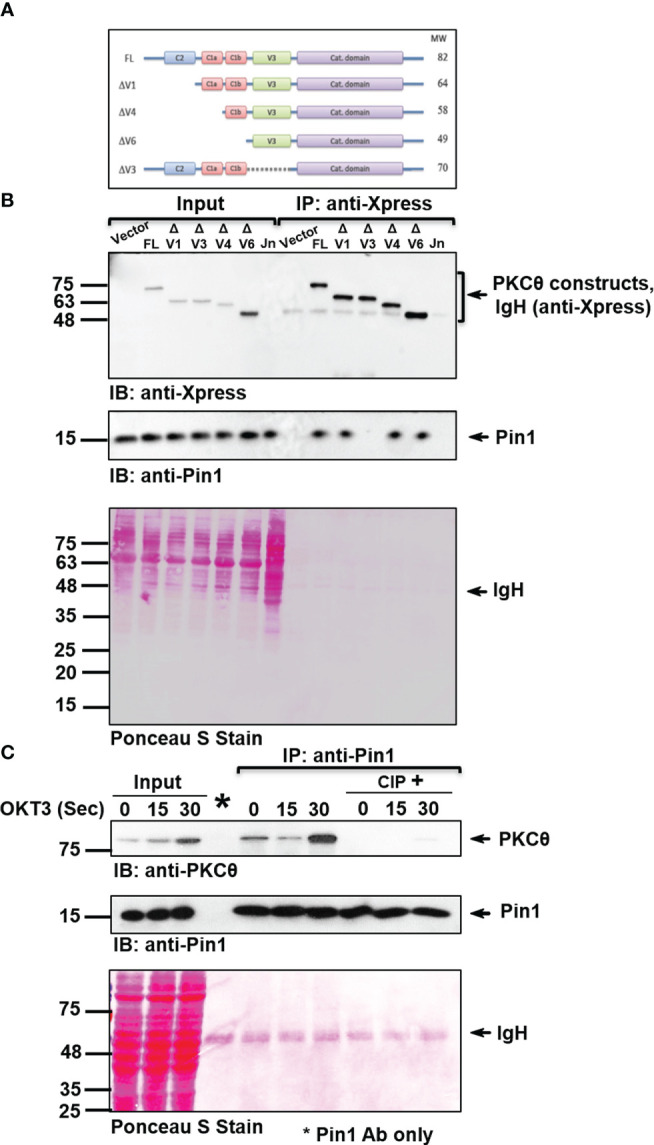
PKCθ-V3 is integral for the phosphorylation-dependent binding of Pin1. **(A)** A schematic representation of Xpress-tagged wild-type or mutant PKCθ constructs. **(B)** Immunoblot (IB) analysis of Xpress (PKCθ) IP or whole cell lysates from COS-7 cells transfected with an empty vector or the indicated Xpress-tagged PKCθ vectors. Untransfected Jurkat E6.1 (Jn) T cells served as a negative control. **(C)** Jurkat TAg cells were stimulated for the indicated time intervals with anti-CD3 mAb (OKT3). Immunoprecipitates (IP) were treated with or without calf intestinal phosphatase (CIP). Following incubation, the samples were subjected to immunoblotting (IB) as indicated. Membrane staining with Ponceau S monitored the equal loading of proteins in all lanes. Data are representative of three independent experiments. Molecular weight markers (in kDa) are indicated on the left and arrow mark positions of the indicated protein bands. IgH, Ig heavy chain.

Based on our preliminary observations, we hypothesized that the phosphorylation of Thr^335^-Pro would turn it into a presumed binding site for the Pin1 enzyme, which specifically recognizes phospho-S/T-P motifs. To determine individual domains of PKCθ that can bind Pin1, we used a heterologous cell system involving COS-7 cells transfected with different Xpress-tagged PKCθ truncation mutant vectors (**Figure 2A**) and tested their ability to coimmunoprecipitate Pin1. Following overexpression of the constructs listed in **Figure 2A**, an anti-Xpress mAb was used for protein immunoprecipitation from the lysates of the transfected COS-7 cells, followed by immunoblot with Pin1-specific mAbs. This experiment unraveled an important observation that the integrity of V3 region is essential for PKCθ in its ability to interact with the Pin1 protein (**Figure 2B**).

PKCθ is subjected to post-translational modifications, primarily by phosphorylation of multiple sites (25). To study whether Pin1 interaction with PKCθ is dependent on phosphorylation, Pin1 was immunoprecipitated from Jurkat T cell lysates and the immunoprecipitate was subjected to treatment with and without enzymatically active phosphatases, followed by gel electrophoresis and immunoblotting. We found that PKCθ that coimmunoprecipitated with anti-Pin1 mAb almost completely dissociated from PKCθ following the treatment with calf intestinal phosphatase (CIP) (**Figure 2C**) or λ protein phosphatase (λPP) (**Figure S1A**). The results indicate that interaction between the two proteins is phosphorylation-dependent. Finally, by utilizing an appropriate isotype control, we validated the specificity of PKCθ coimmunoprecipitation with Pin1 (**Figure S1B**).

### Pin1-PKCθ interaction is mediated by the Pin1 N-terminal WW domain and the PKCθ pThr^335^-Pro motif

**Figure 3:**
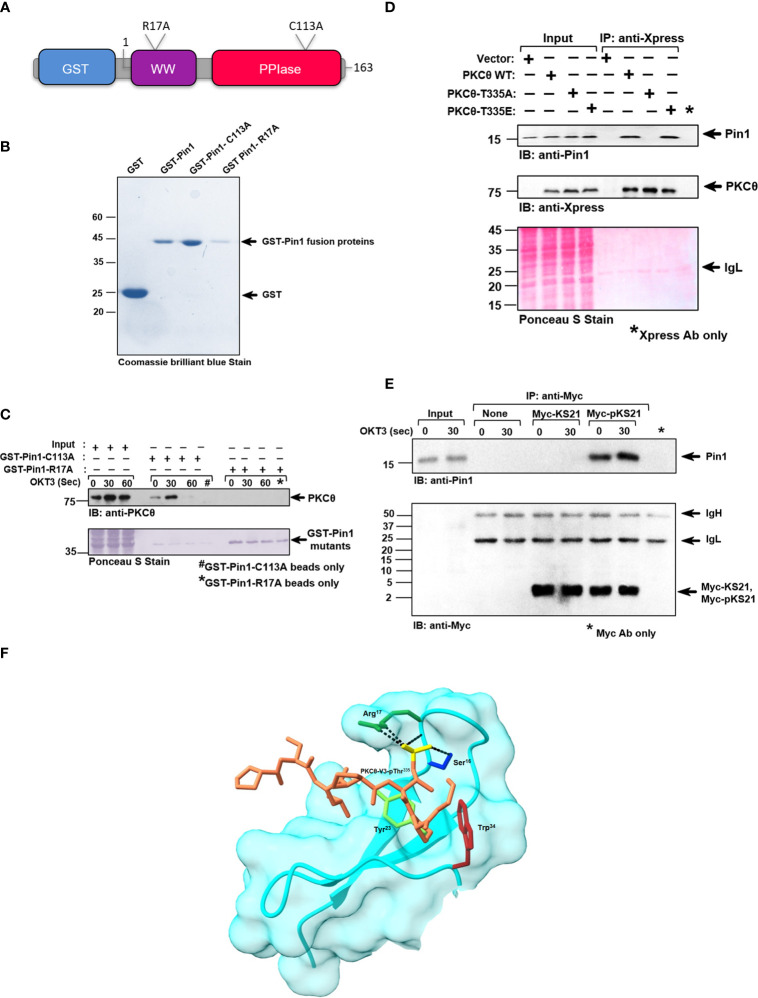
Pin1-PKCθ association is mediated by the N-terminal WW domain of Pin1 and the pThr^335^-Pro motif in PKCθ. **(A)** Schematic representation of GST-tagged point mutants of Pin1 utilized for this study, as indicated. The position and nature of the amino acid substitutions are indicated. **(B)** Coomassie brilliant blue stain of DH5α-expressed, purified GST-tagged wildtype and point mutants of Pin1 protein, utilized in this study. **(C)** Jurkat TAg cells were stimulated with anti-CD3 mAb (OKT3) at the indicated intervals and cell lysates were subjected to a pull-down assay using GST-Pin1-R^17^A and GST-Pin1-C^113^A fusion proteins followed by immunoblot (IB), as indicated. **(D)** Immunoblot (IB) analysis of Xpress (PKCθ) immunoprecipitates from COS-7 cells transfected with an empty vector or the indicated Xpress-tagged PKCθ vectors. Data are representative of three independent experiments. IgH, Ig heavy chain. **(E)** Myc-tagged peptides, KS21 and pKS21, which correspond to Thr335, in the PKCθ-V3 domain, plus ten amino acids on either side of this sequence. KS21-Myc peptide lacks a phosphoryl group on Thr335 while pKS21-Myc, consists of an additional phosphoryl group on the Thr335 motif. Peptides (5 μg/sample) were conjugated with protein A-agarose bead-immobilized anti-Myc Abs. Jurkat TAg cells were left unstimulated or stimulated for 30 sec with anti-CD3 mAb (OKT3). Myc-KS21 and Myc-pKS21 were then immunoprecipitated (IP) from the cell lysates using anti-Myc mAbs. Whole-cell lysates (Input) and immunoprecipitates were subjected to PAGE on 16.5% Tris-tricine gel followed by sequential immunoblot (IB) with anti-Pin1 and anti-Myc Abs. Data are representative of three independent experiments. Molecular weight markers (in kDa) are indicated on the left and arrow mark positions of the indicated protein bands. IgH, Ig heavy chain. **(F)** A model of the PKCθ-Pin1 complex, provides a structural interpretation of some key experimental observations. The surface and backbone of Pin1’s WW domain are depicted in cyan. Four Pin1 residues are presented in atomic detail: Ser^16^ (blue), Arg^17^ (dark green), Tyr^23^ (light green), and Trp^34^ (maroon). The atoms, of the PKCθ-V3-derived short peptide, are colored brown with the phosphate group depicted in yellow. The packing of Tyr^23^ and Trp^34^ with the proline ring, and the hydrogen-bonds/salt bridges of Ser^16^ and Arg^17^ with the phosphate group (black dashed lines) explain the essential role of these residues in peptide binding, and the pSer/Thr-Pro specificity of the WW domain. The sparse interactions with the rest of the peptide explains the promiscuity of the WW domain with respect to the rest of the peptide sequence.

Pin1 has a modular domain architecture comprising an N-terminal WW domain (amino acids 6-37), a flexible linker (amino acids 38-53), and a C-terminal parvulin-type catalytic PPIase domain (amino acids 54-163) (**Figure 3A**). The WW domain is responsible for Pin1’s binding to pSer/Thr-Pro motifs in substrate proteins, while the PPIase domain catalyzes the interconversion of the *cis* and *trans* isomers of proline amide bonds. Both domains recognize pS/T-P motifs (26). A substitution mutation, R^17^A, in the WW domain of Pin1 abolishes the ability of Pin1 to interact with its known substrates, whereas a substitution mutation, C^113^A, downregulates the Pin1 catalytic activity. To determine the Pin1 domain that interacts with PKCθ we used bead-immobilized GST fusion proteins that include Pin1 without or with substitution replacement in the WW (R^17^A) or catalytic (C^113^A) domains (**Figure 3B**). Using a lysate of TCR-stimulated Jurkat T cells in a pull-down assay we found that mutation in R17, but not in C113, abolished the ability of Pin1 to bind PKCθ (**Figure 3C**). The data indicate that the integrity of the WW domain, but not the PPIase domain, is essential for Pin1-PKCθ interaction in T cells.

Since we showed that PKCθ-V3 is required for Pin1 binding (**Figure 1**), we extended our studies on whether the Thr^335^-Pro motif within PKCθ-V3 could be integral for the binding of Pin1. Hence, we conducted a study in COS-7 cells that were transfected with PKCθ WT, and mutants T^335^A, and T^335^E, immunoprecipitated the transiently expressed PKCθ from their lysates (using anti-Xpress mAbs) and tested for Pin1 coimmunoprecipitation. As expected, Pin1 coimmunoprecipitated with PKCθ from the lysate of COS-7 cells transfected with PKCθ WT. However, replacement of Thr^335^ by Ala completely abolished the ability of PKCθ to bind Pin1 whereas substitution of Thr^335^ by the Glu phosphomimetic, reinstated Pin1 binding (**Figure 3D**). The data shows that phosphorylation of the Thr^335^-Pro motif in the PKCθ-V3 is critical for Pin1 binding. We put forth an alternate approach to corroborate our observations by using two Myc-tagged 21-mer peptides, KS21, and pKS21, which correspond to a PKCθ-V3-derived amino acid sequence that includes Thr^335^ plus 10 adjacent amino acids on each side. While both peptides had identical sequences, only pKS21-Myc was phosphorylated on Thr^335^. When each of the two peptides was added to a lysate of human Jurkat T cells, both peptides were detected following immunoprecipitation with anti-Myc mAbs. However, coimmunoprecipitation of Pin1 occurred only in the presence of pKS21-Myc, corroborating the requirement for phosphorylation of PKCθ-V3 on the Thr^335^-Pro motif to turn PKCθ into a Pin1 binding partner (**Figure 3E**). In addition, *in silico* structural modeling analysis revealed a suggestive interaction between PKCθ-Thr^335^-Pro and the critical amino acid residues in the Pin1-WW binding domain (Arg^17^, Tyr^23^, and Trp^34^) to materialize a PKCθ-Pin1 complex (**Figure 3F** and **Figure SV1**). Overall, these results demonstrate that Pin1-PKCθ interaction is mediated by the N-terminal WW domain of Pin1 and the PKCθ-pThr^335^-Pro motif.

### T cell activation-induced binding of Pin1 to PKCθ in C57BL/6J-derived splenocytes and Jurkat T cells follows a temporal time kinetic, whereas interaction is constitutive in mouse thymocytes

**Figure 4:**
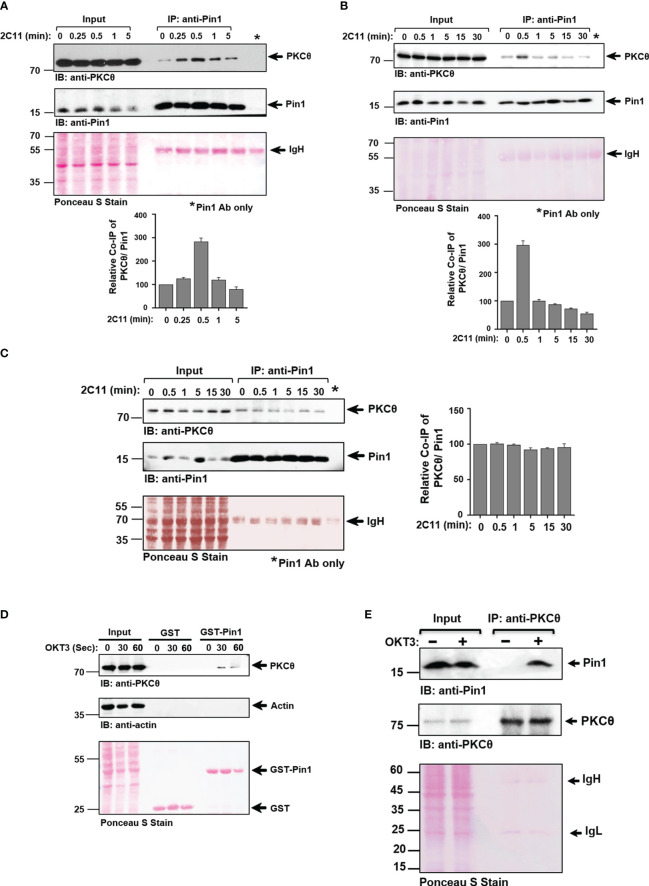
Pin1 association with PKCθ in C57BL/6J-derived T cells is inducible and cell activation-dependent in splenic T cells, in contrast to the constitutive association in thymic T cells. A-B. Splenic T cells derived from C57BL/6J mice were activated for the indicated time points [0-5 min **(A)**], or [0-30 min **(B)**] using anti-CD3ϵ mAbs (2C11). Whole-cell lysates (Input) and Pin1 immunoprecipitates (IP) were subjected to immunoblotting (IB), as indicated, and quantification of the protein band signal intensities was performed using Image J software. **(C)** Thymic T cells derived from C57BL/6J mice were activated for the indicated time points (0-30 min) using anti-CD3ϵ mAb (2C11). Whole-cell lysates (Input) and Pin1 immunoprecipitates (IP) were subjected to immunoblotting (IB), as indicated, and the protein band signal intensities was performed using Image J software. **(D)** Jurkat TAg cells were stimulated with anti-CD3 mAb (OKT3) at the indicated time intervals and cell lysates were subjected to a pull-down assay using GST and GST-Pin1 fusion proteins followed by immunoblotting (IB), as indicated. **(E)** OKT3-stimulated Jurkat T cells were subjected to immunoprecipitation using anti-PKCθ mAbs, followed by SDS-PAGE of WCL (Input) and PKCθ immunoprecipitates and sequential immunoblotting with anti-Pin1 and anti-PKCθ mAbs. Membrane staining with Ponceau S monitored the equal loading of proteins in all lanes. Data are representative of three independent experiments. Molecular weight markers (in kDa) are indicated on the left and arrow mark positions of the indicated protein bands. IgH, Ig heavy chain.

To further analyze the ability of Pin1 to interact with PKCθ in T cells, we used Jurkat T cells and mouse primary cells derived *ex vivo* from C57BL/6J mouse thymus and spleen. The cells were stimulated by crosslinking their TCRs using OKT3 (Jurkat T cells) and 2C11 (splenic/thymic cells) mAbs and the lysates were subjected to Pin1 immunoprecipitation and tested for PKCθ coimmunoprecipitation. We found that PKCθ coimmunoprecipitated with Pin1 from TCR/CD3-stimulated splenic cells (**Figures 4A, B**) and Jurkat cells (**Figure S2A**) with a maximal association occurring at ~30 secs post-TCR stimulation. In contrast, in thymic cells, PKCθ interacted with Pin1 at basal level at all time points post-TCR-crosslinking (**Figure 4C**). The results show that association of Pin1 with PKCθ in TCR-triggered splenic T cells and Jurkat T cells occurs in a time kinetic which corresponds to that of the recruitment of PKCθ to the IS. The constitutive binding of Pin1 to PKCθ that was observed in thymocytes likely represents the ongoing exposure of a large proportions of the cells to signals leading to proliferation, expansion, differentiation and apoptosis during the negative and positive thymic selection processes. Pin1-PKCθ interaction was further validated by demonstrating the ability of GST-Pin1 to pull down PKCθ from TCR-stimulated Jurkat T cells (**Figure 4D**). We also analyzed the selectivity of the Pin1 PPIase to PKCθ by comparing the ability of other PPIases (CypA and FKBP12) to associate with it. Indeed, we found that Jurkat T cell-derived PKCθ selectively associates with Pin1 but not with other peptidyl-prolyl *cis-trans* isomerases (**Figure S2B**). Finally, we found that Pin1-PKCθ association can also be demonstrated using a reciprocal immunoprecipitation, where anti-PKCθ mAbs coimmunoprecipitate Pin1 from lysates of OKT3-stimulated Jurkat cells (**Figure 4E**).

### TCR engagement promotes PKCθ and Pin1 clustering at the plasma membrane within the immunological synapse

**Figure 5:**
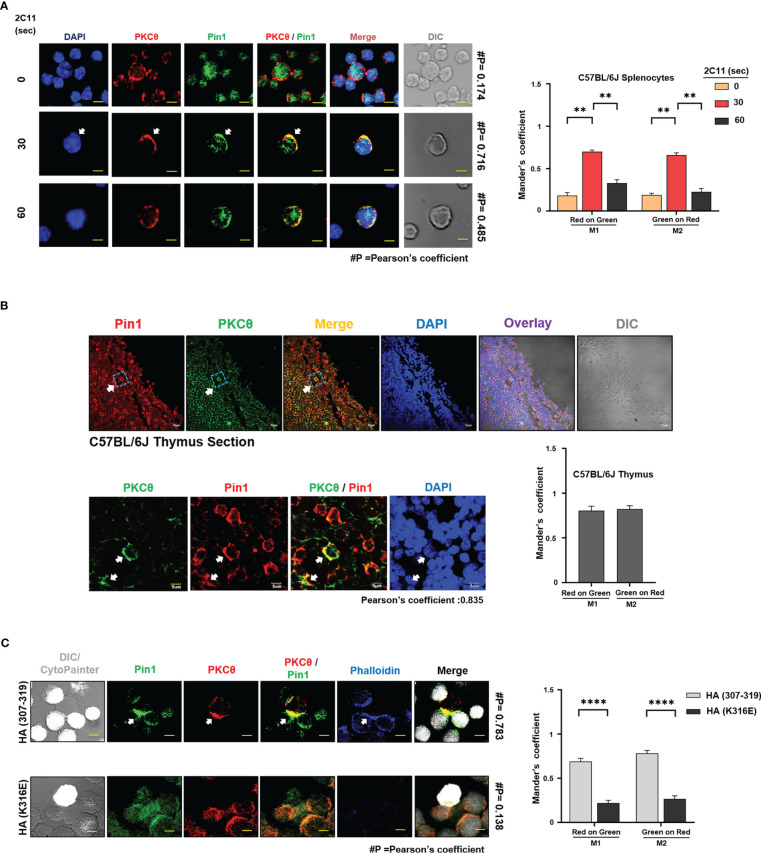
TCR engagement promotes PKCθ and Pin1 clustering at the plasma membrane within the immunological synapse. **(A)** Confocal laser microscopy analysis of Pin1 and PKCθ distribution in 2C11-stimulated C57BL/6J-derived splenic T cells. Arrow indicates Pin1-PKCθ colocalization. Scale bar, 5μm. Quantification of red (PKCθ) and green (Pin1) colocalization was assessed using the JACoP plugin from ImageJ. #P indicates the Pearson’s colocalization coefficient. Mander’s coefficients indicate the fraction of red overlapping with green (M1) and green overlapping with red (M2), respectively. Mean ± SEM (n=9), **P<0.001 (one-way ANOVA, Tukey’s Multiple Comparison Test) **(B)** Confocal laser microscopy analysis of Pin1 and PKCθ distribution in C57BL/6J mouse thymic sections. Arrows indicate Pin1-PKCθ colocalization. Scale bar, 10μm (inset); 5μm (zoom). Quantification of red (Pin1) and green (PKCθ) colocalization was assessed using the JACoP plugin from ImageJ. Pearson’s colocalization coefficient is indicated below the Image. Mander’s coefficients indicate the fraction of red overlapping with green (M1) and green overlapping with red (M2), respectively, and is represented in a bar graph. Mean ± SEM (n=9). **(C)** Conjugate formation between Jurkat T cells, clone CH7C17, and APCs (derived from the LG2 B cell line). Following antigen **(**HA peptide 307-319, or an inactive HA peptide, HA-K316E**)** pre-pulsing and coincubation for 5 min, T cells and APCs were fixed, permeabilized, stained and analyzed by confocal microscopy (scale bar equals 2 μm). Immunofluorescence staining was performed using primary Abs; mouse anti-PKCθ, and rabbit anti-Pin1, followed by Alexa Fluor 633-conjugated phalloidin and secondary Abs, Alexa Fluor 488-conjugated anti-mouse IgG Abs and Alexa Fluor 546-conjugated anti-rabbit IgG Abs. CytoPainter stain was detected at 405nm wavelength. Quantification of red (PKCθ) and green (Pin1) colocalization was assessed using the JACoP plugin from ImageJ. Mander’s coefficients indicate the fraction of red overlapping with green (M1) and green overlapping with red (M2), respectively. Mean ± SEM (n=9), ****P<0.0001 (ordinary two-way ANOVA). DIC, Differential interference contrast. Results are representative of three independent experiments.

PKCθ reside in the cytosol of resting T cells and translocate to the cell membrane upon activation (2) while majority of the Pin1 proteins in resting T cells are confined to the cell nucleus (27, 28). To define the subcellular location of PKCθ-bound Pin1 in TCR-activated T cells, mouse spleen and Jurkat T cells were stimulated with anti-CD3 (2C11/OKT3) mAbs for 30 seconds, followed by staining with fluorescently-labeled Pin1- and PKCθ-specific Abs. Cell analysis using confocal microscopy demonstrated an augmented association between Pin1 and PKCθ at the cell membrane (**Figure 5A**, and **Figure S3A**). Previous observations showing a constitutive association between Pin1 and PKCθ in mouse thymocytes prompted us to analyze the distribution of the same by staining of C57BL/6J thymic sections. Immunofluorescence staining and imaging analysis revealed a strong association between Pin1 and PKCθ at the membrane of thymocytes in the tissue section (**Figure 5B**). Control experiments, utilizing a fluorescently-labeled mouse, and rabbit secondary Abs, excluded the possibility of a basal level or non-specific staining of Pin1 and PKCθ (**Figure S3B**). The results corroborate the interaction of Pin1 and PKCθ at the membrane of T cells and hint for a possible involvement of Pin1 in the regulation of PKCθ and PKCθ-dependent early activation events in TCR-engaged T cells.

Since engagement of the TCR promotes PKCθ translocation to the center of the IS, we further analyzed whether the same applies for the PKCθ-bound Pin1 protein. We used Jurkat T cells (clone CH7C17), which express the influenza hemagglutinin (HA) peptide-specific Vβ3 TCR, and co-cultured them with antigen-fed LG2 B cells, as APC. Cell staining with fluorescently-labeled Pin1- and PKCθ-specific Abs and analysis using a confocal microscope we observed that in a fraction of antigen (HA peptide 307-319) -pulsed LG2 cells that formed conjugates with the CH7C17 T cells, Pin1 and PKCθ colocalized at the T cell-APC contact area (**Figure 5C**). The colocalization of Pin1 and PKCθ was antigen-specific since it failed to occur when LG2 cells were fed with a mutated HA peptide possessing a single amino acid substitution (HA-K316E). The F-actin binding protein, phalloidin, served as an IS-specific marker in this study.

## Discussion

In the past three decades, the role of PKCθ in T cells was intensively studied. It was initiated in 1993, when the human PKCθ gene was originally cloned (29), yielding many important findings along these years (30–32). The present work focuses on the Thr^335^-Pro motif within the PKCθ-V3 regulatory domain and its identification as a key priming site for the IS localization and activation of PKCθ. Moreover, we demonstrated that Thr^335^-Pro could serve as a docking site for the Pin1 enzyme, which specifically recognizes phospho-Ser/Thr-Pro motifs (10, 33).

PKCθ is subjected to allosteric regulation and post-translational modifications, primarily by phosphorylation at multiple sites (25). For example, phosphorylation of Thr^538^ at the activation loop (34), is mediated by the GLK kinase (35) and is critical for PKCθ activation and TCR downstream signaling (25). The role of other potential phosphorylation sites is not fully understood. Recently, a phospho-Ser^241^-Pro-Thr motif in PKCθ was predicted to function as a nuclear localization signal (NLS) (36), while our present results suggest that Thr phosphorylation within the Thr^335^-Pro-Gly motif is required for the IS localization and activation of PKCθ. The selective localization of the PKCθ isoform is mediated by a direct or indirect association between its V3 domain and the cytoplasmic tail of CD28, the prototypic costimulatory receptor on T cells. This interaction is mediated by the Lck protein tyrosine kinase (PTK) that associates, *via* its SH2 and SH3 domains, with a tyrosine-phosphorylated CD28 motif and a PR sequence in the PKCθ V3 region, respectively (9). Here, we elucidate, for the first time, the mechanistic basis for the selective, non-redundant IS localization and function of PKCθ in T cells.

Herein, we demonstrate that Thr^335^-Pro in the PR motif within the PKCθ-V3 domain is a TCR-stimulation-dependent phosphorylation site which is critical for PKCθ recruitment and localization at the IS, a prerequisite for the induction of TCR-linked signal transduction pathways. Substitution of Thr^335^ by Ala completely abolished PKCθ activation and its ability to bind CD28, while Thr^335^ replacement by Glu, which mimics the approximate size and charge of phospho-Thr, restored these properties. Fluorescent cell staining and confocal microscopy confirmed that phospho-Thr^335^-Pro is required for IS localization of PKCθ. Our studies highlight the potential role of the Thr^335^-Pro motif in the spatiotemporal regulation of PKCθ and corroborate the assumption that regulation *via* this motif is phosphorylation-dependent.

Furthermore, we highlight phospho-Thr^335^-Pro as a putative site for association with the Pin1 PPIase. Pin1 association with PKCθ in TCR-triggered T cells is dependent on the phosphorylation state of PKCθ. Interaction between these two proteins is hampered by the presence of active phosphatases (CIP and λ PP), supporting the assumption that phosphorylated Ser/Thr residues in PKCθ are required for the interaction with Pin1.

Pin1 possesses two major functional domains; an N-terminal substrate-binding WW domain (amino acids 6-37) and a C-terminal PPIase catalytic domain (amino acids 54-163). We found that a single amino acid substitution mutation, R^17^A, in the WW domain of Pin1 abolished the ability of Pin1 to interact with PKCθ. In contrast, a substitution mutation, C^113^A, which downregulates the Pin1 catalytic activity had no effect on its ability to interact with PKCθ. Therefore, it appears that the integrity of the WW domain, and not the catalytic domain of Pin1 is essential for interaction with PKCθ in activated T cells.

Based on previous studies, showing that the PKCθ Thr^335^-Pro-Gly motif is required for the activation and association of PKCθ with CD28, we hypothesized that the phospho-Thr^335^-Pro-Gly sequence might serve as a binding site for Pin1. Utilizing a heterologous system of COS-7 cells that were transfected with wild type PKCθ, or PKCθ mutants we found that the Thr^335^-Pro-Gly motif of PKCθ is essential for Pin1 binding. The utilization of PKCθ-deficient COS-7 cells eliminated the influence of endogenous PKCθ in the mutant protein coimmunoprecipitation studies. A plausible structural interpretation of this observation is supported by a computational model of the PKCθ-V3-derived short peptide, in association with the Pin1-WW domain.

Previous studies demonstrated that Pin1 can interact with and downregulate the activity of cPKC isoforms (18). In these studies, COS-7 cells were cotransfected with the relevant PKC isoform plus GST-Pin1, and PKC-Pin1 binding was evaluated by GST pull down and immunoblot. In contrast to the cPKC isoforms that were easily detected in the pull-down assay, only a small fraction of the cellular PKCθ was pulled down by GST-Pin1. In the present study, we pulled down PKCθ from Jurkat T cell lysates using bead-immobilized GST-Pin1 and Pin1 mutants and observed a relatively strong association between the two proteins. The *in vivo* association between PKCθ and Pin1 was further substantiated using mouse splenic T cells as well as by coimmunoprecipitation and cell staining analyses. Although the Pin1 isomerization site in the cPKC isoforms is missing in PKCθ (18), we found that PKCθ possesses a distinct Pin1 binding site within its regulatory regions, which is absent in cPKC isoforms.

The current binding studies performed in T cells from different sources, including human Jurkat T cells and freshly isolated mouse splenic T cells, revealed a transient association between PKCθ and Pin1 that was augmented following TCR crosslinking. In contrast, a cell activation-independent constitutive association between PKCθ and Pin1 was observed in thymic T cells, which include a high proportion of proliferating and differentiating cells that undergo positive and negative selection in response to triggers by thymic epithelial cells and the thymic microenvironment. A constitutive interaction between Pin1 and PKCθ was also observed in fluorescently labeled thymocytes in thymic sections derived from C57BL/6J mice. The thymus consists of heterogeneous cell populations that upregulate distinct kinase activities during different stages of differentiation and maturation (37–39), and at a certain stage (CD4^+^CD8^+^ double positive cells) also form multifocal immunological synapse with sustain tyrosine phosphorylation (40). While functionally active PKCθ is known to be essential for the positive selection of thymocytes (41), expression of functinal Pin1 in thymocytes appears to be as important, since its ablation disrupts their genetically predetermined mechanisms of differentiation and maturation (42).

The rapid and transient interaction between PKCθ and Pin1 follows a time kinetic that corresponds to that of PKCθ recruitment to the IS, suggesting that Pin1 might play a role in the regulation of PKCθ recruitment to the IS, and in activation of the PKCθ-dependent early activation events in TCR-triggered T cells. Members of each of the three families of PPIase (cyclophilins, FKBPs, and parvulins) regulate the conformation, stability, localization, and activity of distinct protein kinases (43–46). We demonstrated that PKCθ interacts with Pin1, but not other PPIases (CypA or FKBP12) in TCR-stimulated T cells, demonstrating the selectivity and specificity of Pin1 to PKCθ.

In resting T cells, PKCθ resides predominantly in the cytosol while majority of the Pin1 proteins are confined to the nucleus (2, 27, 28). Immunofluorescence imaging of TCR/CD3-activated Jurkat T cells showed enhanced recruitment of Pin1 and PKCθ to the plasma membrane, where the two proteins colocalized, providing further support for the *in vitro* results showing reciprocal coimmunoprecipitation of the two proteins. Strong colocalization of Pin1-PKCθ was visualized at the heterotypic cell contact area, and since PKCθ is known to localize to the center of the IS, the results suggest that Pin1 in TCR-activated T cells is an integral constituent of the IS. To further support the hypothesis that Pin1 colocalize with PKCθ at the IS we utilized antigen-specific T cells and induced their activation using antigen-fed APC. This system included Jurkat T cells, clone CH7C17, which express a Vβ3 TCR specific for the influenza HA 307-319 peptide, and LG2 B cells, as APC, that were fed with the specific HA peptide or with a mutated peptide that possess a single amino acid substitution (HA-K316E). Coincubation of the two cell types followed by their staining with fluorescently-labeled Pin1- and PKCθ-specific Abs and analysis using a confocal microscope revealed that in a fraction of cells that formed conjugates, Pin1 and PKCθ colocalized at the IS, at the T cell-APC contact area, that was co-stained by the fluorescently-labelled F-actin binding protein, phalloidin, which serve as an IS-specific marker. The results highlight the possibility that Pin1 functions as a molecular timer that finetunes PKCθ phosphorylation and activation and contributes to the regulation of its downstream effector molecules.

Previous studies demonstrated that PKCθ IS localization requires the interaction of PKCθ with the cytoplasmic tail of CD28 (9, 31). This indirect interaction is made possible by Lck which utilizes its SH2 and SH3 domains for the simultaneous interaction with a PYAP motif in CD28 and a PxxP motif in PKCθ, respectively. The sites of interaction of Lck (P^331^CLP) and Pin1 (T^335^PG) in PKCθ are extremely close, suggesting a potential binding competition between Pin1 and Lck. However, Pin1 and Lck might also interact with PKCθ independent of each other, or in a sequential manner. For example, Pin1 interaction with PKCθ might define the conformation of the latter, which will determine the accessibility of the PKCθ P^331^xxP motif for interaction with Lck, and consequently, the ability of the Lck-PKCθ complex to recruit to the IS. Furthermore, the competitive, independent, or sequential binding of Pin1 and Lck to PKCθ may differ among T cell subtypes and thereby explain the differential recruitment of PKCθ to the IS of Th/Tc vs its recruitment to the opposite cell pole in Treg.

Preliminary studies in Pin1 shRNA-expressing Jurkat T cells revealed that knock-down of Pin1 downregulates NF-κB reporter activity in TCR stimulated cells (**Figure S4**). However, since Pin1 regulates the conformation and activity of multiple proteins, these findings could reflect either a direct effect of Pin1 on PKCθ or on PKCθ downstream effectors that regulate the activity of NF-κB.

The current study identified a previously undescribed signature motif in the PKCθ modular structure, which is required for PKCθ IS localization and activation. Furthermore, TCR-stimulation-induced phosphorylation of this motif dictates its ability to interact with Pin1. Together, we report on a novel interaction between two enzymes- PKCθ and Pin1, which might contribute to the amplitude, duration, and fine-tuning of the T cell activation response.

## Data availability statement

The original contributions presented in the study are included in the article/**Supplementary Material**. Further inquiries can be directed to the corresponding author.

## Ethics statement

This study was approved in advance by the Ben-Gurion University Institutional Animal Care and Use Committee and conducted in accordance with the Israeli Animal Welfare Act following the guidelines of the Guide for Care and Use of Laboratory Animals (National Research Council 1996). The Animal ethical clearance protocol used for this research is IL-44-08-2017.

## Author contributions

NI conceived the study, designed experiments, and supervised the project. NPA, AM, PRN, and K-FK carried out the experiments. CK conducted the structural studies. ZS, AB, AA, and NI analyzed the data. EL and AA provided essential tools for the study. NPA, and NI wrote the manuscript. All authors commented on the previous versions of the manuscript. All authors contributed to the article and approved the submitted version.
