# Supplementary material for: The Peptidyl-Prolyl cis-trans isomerase, Pin1, associates with Protein Kinase C θ via a critical Phospho-Thr-Pro motif in the V3 regulatory domain
Source: Front Immunol. 2023 Mar 8;14:1126464. doi: 10.3389/fimmu.2023.1126464 (PMC10031136; doi:10.3389/fimmu.2023.1126464)
Supplement: Supplementary file 1 [file DataSheet_1.docx]

**SUPPLEMENTARY INFORMATION**

**Figure S1**

**
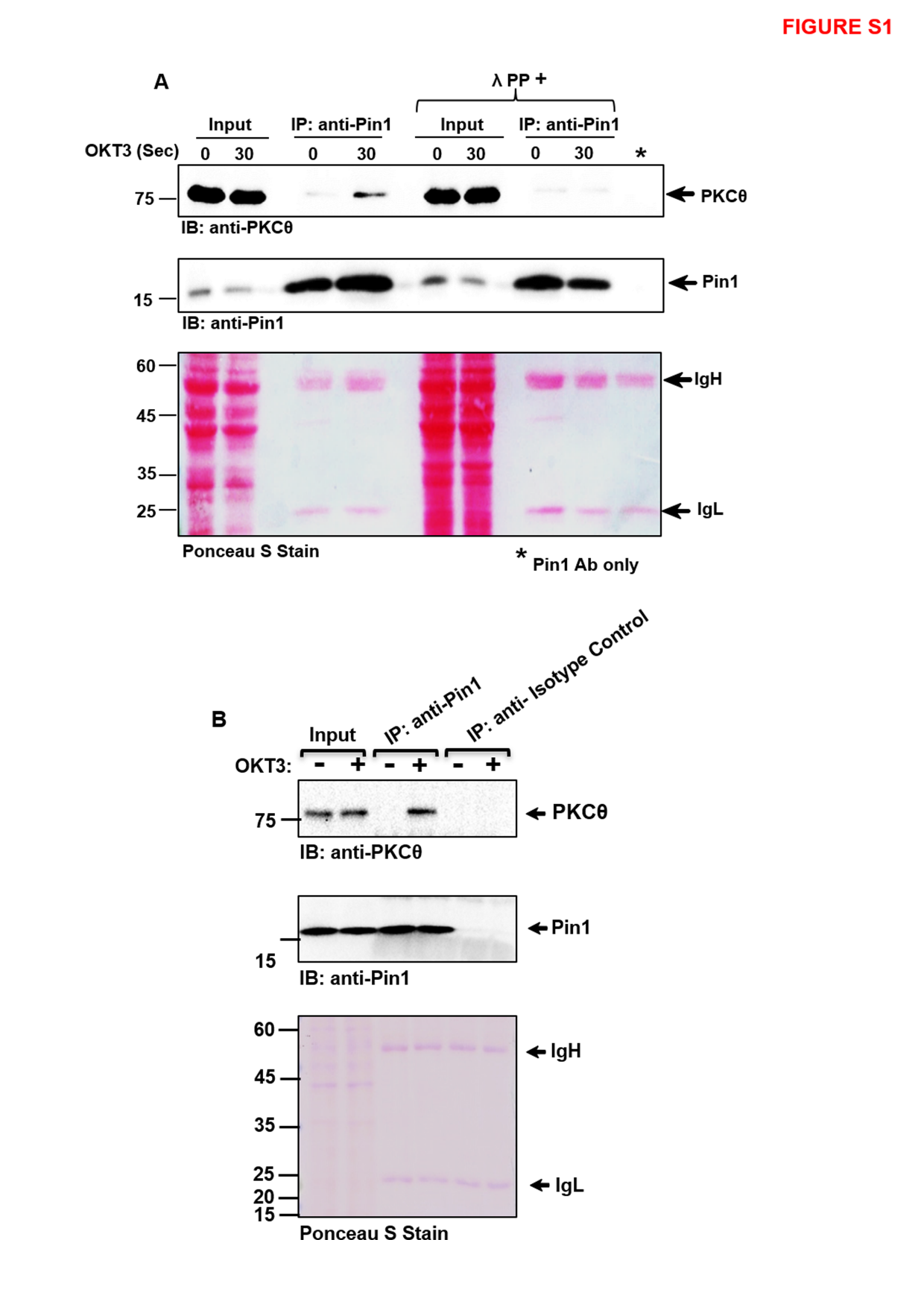
**

**Figure S2**

**
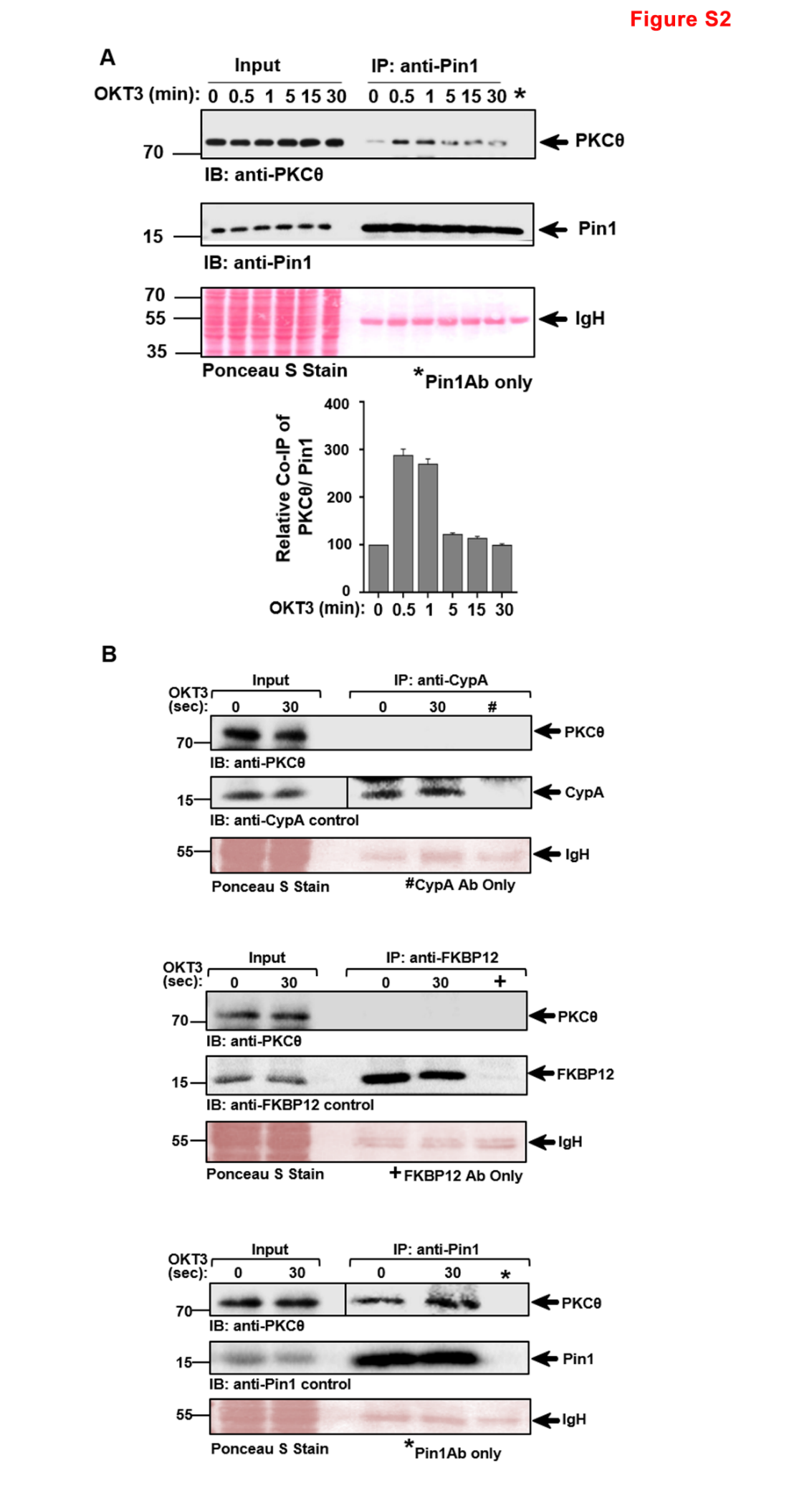
**

**Figure S3**

**
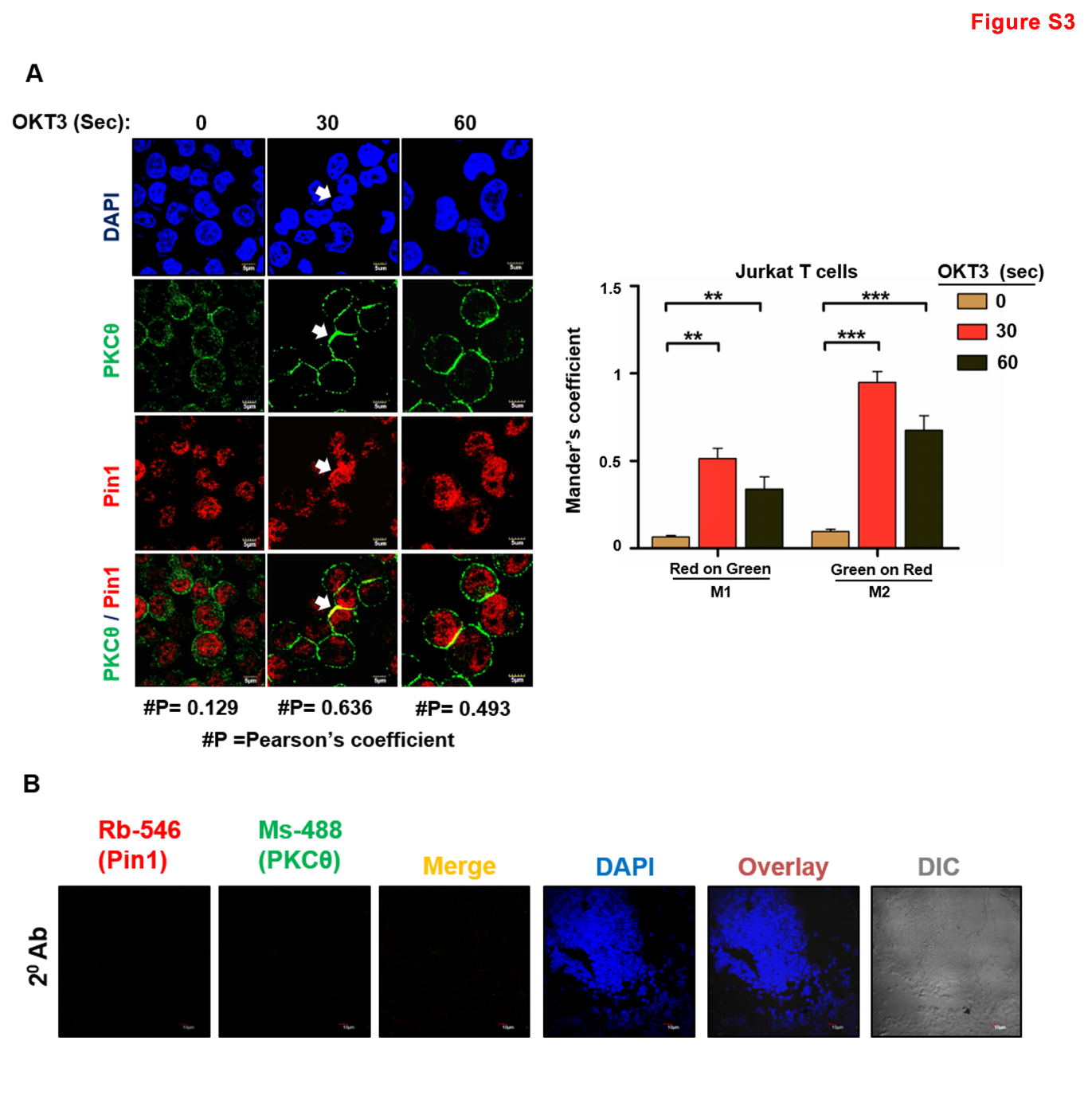
**

**Figure S4**

**FIGURE LEGENDS**

**Figure S1. Pin1-PKCθ complexes undergo dissociation in the presence of protein phosphatases. A.** Jurkat TAg cells were stimulated for the indicated time intervals with anti-CD3 mAb (OKT3). Whole-cell lysates (WCL; Input), and immunoprecipitates (IP) were treated with λ-phosphatase (λ PP). Following incubation, the samples were subjected to immunoblotting (IB) as indicated. Membrane staining with Ponceau S monitored the equal loading of proteins in all lanes. Molecular weight markers (in kDa) are indicated on the left and arrows mark the position of the indicated protein bands. IgH, Ig heavy chain; IgL, Ig light chain. Results are representative of three independent experiments. **B.** Jurkat T cells were stimulated with OKT3 mAbs for 1 min. Cell lysates were then subjected to immunoprecipitation using rabbit anti-Pin1 and rabbit anti-IgG isotype control mAbs. The samples were then subjected to SDS-PAGE and sequential immunoblotting with anti-PKCθ and anti-Pin1 mAbs. Membrane staining with Ponceau S monitored the equal loading of proteins in all lanes. Molecular weight markers (in kDa) are indicated on the left and arrows mark the position of the indicated protein bands. Results are representative of three independent experiments. IgH, Ig heavy chain; IgL, Ig light chain.

**Figure S2. T cell activation-induced binding of Pin1 to PKCθ in Jurkat T cells is selective and transient. A.** Jurkat TAg cells were stimulated for the indicated time intervals (0-30 min) with anti-CD3 mAbs (OKT3). Whole-cell lysates (Input) and Pin1 immunoprecipitates (IP) were subjected to immunoblotting (IB) as indicated. **B.** Comparative analysis of the association of PKCθ with representatives of the three different families of PPIases (CypA, FKBP12, and Pin1) in TCR/CD3-triggered Jurkat TAg cells. Membrane staining with Ponceau S monitored the equal loading of proteins in all lanes. Molecular weight markers (in kDa) are indicated on the left and arrows mark the position of the indicated protein bands. Results are representative of three independent experiments. IgH, Ig heavy chain.

**Figure S3. Pin1 colocalizes with PKCθ at the membrane of TCR/CD3-stimulated human Jurkat T cells. A.** Confocal laser microscopy analysis of Pin1 and PKCθ distribution in OKT3-stimulated Jurkat T cells. Arrow indicates Pin1-PKCθ colocalization. Scale bar, 5μm. Quantification of red (Pin1) and green (PKCθ) colocalization was assessed using the JACoP plugin from ImageJ. #P indicates the Pearson's colocalization coefficient. Mander’s coefficients indicate the fraction of red overlapping with green (M1) and green overlapping with red (M2), respectively. Mean+SEM (n=9), ****** P<0.01, *******P<0.001 (one-way ANOVA, Tukey's Multiple Comparison Test). Data are representative of three independent experiments. **B.** A control experiment demonstrating the staining of C57BL/6J mouse thymic sections with fluorescently-labeled Alexa Fluor™546-conjugated goat anti-mouse and Alexa Fluor™488-conjugated goat anti-rabbit secondary Abs, to rule out the possible basal, non-specific staining. Scale bar equals 10 μm.

**Figure S4.** **Pin1 knock-down reduces NF-**κ**B reporter activity in Jurkat T cells.** Jurkat E6.1 cells and Jurkat-derived clones that stably overexpress Pin1-specific short hairpin RNA (shRNA) or scrambled shRNA (scRNA) were co-transfected with NF-κB luciferase reporter plasmid and a *Renilla* luciferase-containing vector. Cells were left unstimulated or stimulated with PMA (100ng/ml) + ionomycin (50 ng/ml) or OKT3 (1:200 dilution of the ascites) + anti-human CD28 (2 µg/ml) for the final 8 hours of the culture. Cells were then lysed using passive lysis buffer and assayed for luciferase activity using the dual luciferase reporter system (Promega). Luminescence values generated by the *Firefly* luciferase were normalized for transfection efficiency using the *Renilla* luciferase activities. The NF-κB-dependent luciferase activity is expressed as fold induction over the values obtained from unstimulated cells.
